# Competitive rational inhibitor design to 4-maleylaceto-acetate isomerase

**DOI:** 10.6026/97320630013140

**Published:** 2017-05-31

**Authors:** Narges Zolfaghari

**Affiliations:** 1 National institute of genetic engineering and biotechnology, Tehran, Iran

**Keywords:** Tyrosinemia type I, Nitisinone, 4- maleylacetoacetate isomerase, rational drug design

## Abstract

Tyrosinemia type I is the result of genetic disorder in fomaryl acetoacetase gene that leads to 4-fumaryl acetoacetate accumulation. The current treatment for tyrosinemia type I is nitisinone that inhibits 4-hydroxyphenyl pyruvic dioxygenase in competitive manner. In the present study, we have designed two theoretical chemicals, which could inhibit the direct enzyme responsible for fumarylacetoacetate formation. Subset 2_p.0.5 from Zinc database was screened by PyRx software using a Lamarckian genetic algorithm as the scoring function for docking. Top nine successive hits were selected for further pharmacological analysis and finally the new designed ligands RD6-2 (3Z)- 1,3-Butadiene-1,1,2,4-tetrol and RD-7-1 ((Z)-3-[4-Hydroxy-1-(hydroxymethyl)cyclohexyl]-2-propene-1,2-diol could pass PhysChem, FAFDrugs and AdmetSAR filter. The designed ligands were non-substrate and non-inhibitor of CYP450 and nontoxic in AMES test. LD50 of RD-6-2 was 793mg/kg with the toxicity class of four and The LD50 of RD-7-1 was calculated as 5000mg/kg within the toxicity class of five. The designed molecules are introduced as the new theoretical small molecules, which can theoretically inhibit 4- maleylacetoacetate isomerase in a competitive manner.

## Background

Tyrosine degradation pathway is one of the key pathways in human physiology, which is associated with several genetic disorders. Several enzymes are engaged in the degradation pathway of tyrosine which any defect of pathogenic mutation in the enzyme genes would cause serious clinical signs. Alkaptonuria is the result of a missense mutation of homogentisate 1,2 dioxygenase gene on chromosome 3q [1]. The clinical result of this mutation is increasing the blood levels of homogentisic acid (HGA) which finally leads to the deposition of pigmented HGA in different tissues of body (specially in cardiovascular system, kidney and skin) [2]. Tyrosinemia type II is the results of missense mutation in tyrosine trans aminase, which is the first enzyme involved in the degradation pathway [3]. Tyrosinemia type III is caused by p- Hydroxyphenyl pyruvate accumulation in the body that is the result of genetical defect in the gene of p-Hydroxyphenyl pyruvate dioxygenase [4]. Moreover, tyrosinemia type I is the result of fumaryl acetoacetase defection and 4-fumaryl acetoacetate accumulation. It is a rare autosomal recessive genetic metabolic disorder and its symptoms appear in the first month of the life including failure to gain weight and grow at the expected rate, fever, diarrhea, vomiting and hepatomegaly [5]. The birth incidence is 1/100,000 for tyrosinemia type 1. The current commercial drug for treating it is nitisinone, which is approved by FDA in 2002 [6]. Nitisinone is a competitive inhibitor of 4-hydroxyphenylpiruvic acid dioxygenase. It decreases the cellular level of Homogentisic acid, an intermediate chemical and precursor of fumarylacetoacetate. In molecular level, for better treatment of tyrosinemia type I it is essential to target a more specific enzyme engaged in fumarylacecoacetate formation. For gaining this purpose, we have tried to design specific chemicals that target 4- maleylacetoacetate isomerase, which is directly responsible for fumarylacecoacetate production. To do this, virtual screening and rational drug design techniques was used.

## Methodology

### Molecular docking analysis

The Crystal structure of human 4- maleylacetoacetate isomerase retrieved from Protein Data Bank (PDB) database (http://www.rcsb.org/pdb/home/home.do) with PDB code of 1fw1 [7]. The structure of 4-fumarylacetoacetate were draw by HyperChem software and optimized by 500 PS molecular dynamics simulation using MM+ force field. Virtual screening library was retrieved from Zinc database [8]. A drug like category subset from Zinc database (2_p.0.5) was downloaded and used as the primary virtual screening library. PyRx software was used for docking operation [9], which is a GUI tool, based on AutoDock and AutoDock vina. The scoring function was Lamarkian genetic algorithm.

### Ligand modification and pharmacokinetic analysis

HyperChem software was used for structural modifications. After each modification process, we have applied 500 PS molecular dynamics simulation to reach the optimal structure. OpernBable GUI tools were also used for format conversion. The rationally designed ligands checked by FAFDrugs (http://fafdrugs3.mti.univ-paris-diderot.fr/) web server [10,11]. The oral toxicity of hits and rationally designed ligands was checked by PROTOX web server (http://tox.charite.de/tox/), which is based on chemical similarities between compounds with known toxic effects and the presence of toxic fragments [12]. In addition, dmetSAR web server (http://lmmd.ecust.edu.cn:8000/) [11] was used to analyze the absorption, distribution, metabolism, and excretion properties of new designed ligands. ADME properties of top successive hits were checked in optimal descriptors (hydrogen bonds, charge) in pH=7.4.

## Results and discussion

**Figure 1:**
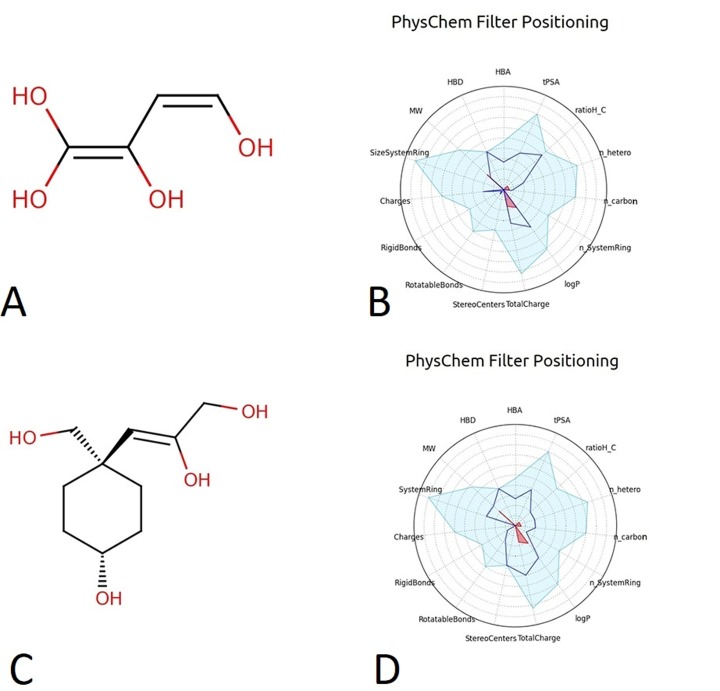
The structural properties of rationally designed ligands. A: The structure of RD-6-2, which is a revised structure based on the successive hit number 6; B: The PhysChem filter indicated that RD-6-2 structure (blue line) in within the lead like are and (blue area); C: The structure of rationally designed ligand RD-7-1, which is a modified structure, based on the successive hit number 7; D: The PhysChem filter result indicated that RD-7-1 structure (blue line) in within the lead like are and (blue area). MW: molecular weight. HBD: hydrogen bonds donor. HBA: hydrogen bond acceptor. tPSA: topological polar surface area. LogP: indicator of hydrophobicity.

In molecular level, production of fumarylacetoacetate should be decreased in order to treat tyrosinemia type 1. There are several options for decreasing fumarylacetoacetate levels available including treatment by Nitisinone that inhibits hydroxyphenylpiruvic acid dioxygenase [13], inhibiting upstream enzymes of tyrosine degradation pathway and inhibiting 4- maleylacetoacetate isomerase by a competitive inhibitor. 4- maleylacetoacetate has two domains: an N-terminal domain containing 84 residues and a C-terminal containing 121 residues. The N-terminal domain contains four beta sheets (7-10, 32-35, 61-64, and 67-70) connected to each other by three alpha helices. The Cterminal domain contains five alpha helix and three 310 helixes. In the present study, we have tried to find a drug like chemical, which could significantly play the role of a competitive inhibitor. For gaining this purpose, a large database was screened against the active site of 4- maleylacetoacetate isomerase in the coordinates of: X: 36.57, Y: 26.09 and Z: 15.24 with a radius of 14 Å to cover the entire active site. Top 100 hits with the best binding efficiency were extracted and analyzed regarding pharmacological properties. Among extracted hits, nine with most lead likeness selected for further pharmacological studies. Table 1 indicates the pharmacological properties of top nine successive hits. Ligand No#2 that could reach the best binding efficiency equals to -11.09 indicated a Lipinski violation in the structure and rejected for further study. In other hands, Lig No#6 and Lig No#7 indicated fewer errors in structure in comparison with other selected hits. Therefore, rational drug design was carried out by these tow structures. The most problem in Lig No#6 structure was the presence of two High Risk aliphatic ketone in C2=O1 and C5=O6 positions. In order to solve this problem, we have changed the ketones to alcoholic groups. Moreover, to prevent ketone formation in the body, we have changed single C2-C4 and C5-C7 to double bonds. It saturated the Carbone free orbitals in positions C2 and C5 which taking part in Ketone formation. The other error in the structure of Lig No#6 was related to C/H ratio, which by changing Ketone to alcohol this problem was solved respectively. The new molecule RD-6-1 was re-analyzed regarding pharmacological properties and just Hydrogen bond donors were remained unfix. In the next step, one alcoholic group at position C2-O1 was removed from the structure. Finally, the new RD-6-2 molecule could pass FAFDrugs as well as PhysChem filters (Figure 1). Moreover, AdmetSAR predictions indicated that RD-6-2 is non-substrate and non-inhibitor of CYP450. In addition, it is predicted to be non-toxic in AMES and carcinogenicity. In other hands, PROTOX prediction indicated that the oral LD50 of RD-6-2 is 793mg/kg with the toxicity class of four (1: most toxic and 6: safe). In addition, no critical human protein target has been found for it.

Lig No#7 was not accepted as a lead like compound due to errors in High-risk hemiketal, stereo centers and Hydrogen bond donors. To remove hemiketal from the structure of Lig No#7, we have removed two alcoholic groups from O17 and O24 positions. Moreover, a single bond changed to double in position C10-C11. It reduced the flexibility and stereo centers of the molecule. Interestingly, RD-7-1 was successfully passed the FAAFDrugs and PhysChem filters. In other hands, the AdmetSAR prediction indicated that the new rationally designed molecule is nonsubstrate and non-inhibitor of CYP450. In addition, RD-7-1 predicted to be not carcinogen and non-toxic in AMES assay. In addition, TROTOX predicted the LD50 of RD-7-1 as 5000mg/kg within the toxicity class of five, which means that it has not oral toxicity. No toxic fragment has been found in the molecule and no critical human body protein binding has been predicted by PROTOX. Both rationally designed chemicals RD-6-2 and RD-7-1 are targeting 4- maleylacetoacetate isomerase, which is the enzyme directly responsible for tyrosinemia type I. in comparison with nitisinone, these two theoretically designed chemicals can be used for treating tyrosinemia type I in a more specific manner.

## Conclusion

Nitisinone decreases 4-fumaryl acetoacetate levels by inhibiting 4- hydroxyphenyl pyruvic dioxygenase, which is not specific enzyme for 4-fumaryl-acetoacetate productions. Two rationally designed molecules RD-6-2 and RD-7-1 that could pass the several pharmacological filters including Lipinski rules, PhysChem, AdmetSAR and FAFDrugs, are introduced as new molecules that can effectively inhibit inhibiting 4- maleylacetoacetate in a competitive manner and as the results, the fumarylacetoacetate levels would be decreased.

## Figures and Tables

**Table 1:** The pharmacological properties of virtual screening and rationally designed ligands. The properties are PhysChem and FAFDrugs filters. HBD: hydrogen bonds donor. HBA: hydrogen bond acceptor. tPSA: topological polar surface area. LogP: indicator of hydrophobicity.

Ligand	Binding	MW	logP	tPSA	Flexibility	HBD	HBA	HBD_HBA	Rings	ratioH/C	Lipinski Violation
RD-6-2	-9.8	118.09	0.28	77.43	0.33	4	4	8	0	1	0
RD-7-1	-9.6	202.25	-0.55	77.76	0.3	4	4	8	1	0.4	0
1	-11.2	156.22	1.67	37.3	0.75	1	2	3	0	0.22	0
2	-11.09	382.62	7.8	17.07	0.15	0	1	1	1	0.04	1
3	-10.9	195.17	-3.42	101.91	0.25	3	5	8	1	0.56	0
4	-10.8	130.1	-0.15	80.26	0.5	2	4	6	0	0.8	0
5	-9.9	130.1	-0.07	80.26	0.4	2	4	6	0	0.8	0
6	-8.56	132.07	-0.63	97.33	0.5	2	5	7	0	1.25	0
7	-11.3	190.11	-1.1	137.46	0.56	3	7	10	0	1.17	0
8	-10.8	226.18	-0.5	117.56	0.31	3	6	9	1	0.6	0
9	-9.7	166.22	2.69	40.13	0.2	1	2	3	1	0.2	0
